# Risk factors for kidney injury during vancomycin and piperacillin/tazobactam administration, including increased odds of injury with combination therapy

**DOI:** 10.1186/s13104-015-1518-9

**Published:** 2015-10-17

**Authors:** Tiffany Kim, Sheetal Kandiah, Manish Patel, Saira Rab, Jordan Wong, Wenqiong Xue, Kirk Easley, Albert M. Anderson

**Affiliations:** Grady Health System Infectious Diseases Program, Atlanta, GA USA; Johns Hopkins Bayview Medical Center, Baltimore, MD USA; Division of Infectious Diseases, Department of Medicine, Emory University School of Medicine, 341 Ponce de Leon Avenue, Atlanta, GA 30308 USA; Biostatistics, Boehringer Ingelheim Pharmaceuticals, Inc., Ridgefield, CT USA; Department of Biostatistics and Bioinformatics, Emory University School of Public Health, Atlanta, GA USA

**Keywords:** Vancomycin, Piperacillin, Acute kidney injury

## Abstract

**Background:**

Acute kidney injury (AKI) occurs frequently in hospitalized patients and has been associated with the administration of certain medications. Concerns have been raised in recent reports that the antibiotic combination of vancomycin and piperacillin/tazobactam (combV/P) may be more associated with AKI than monotherapy with either drug.

**Methods:**

To compare the incidence of and risk factors for AKI in patients receiving combV/P versus monotherapy with either drug, a retrospective study was conducted in non-critically ill inpatients at a large urban teaching hospital. AKI was defined as either: (1) Increase in serum creatinine ≥0.5 mg/dl OR (2) ≥1.5-fold creatinine increase from admission baseline. In addition to standard multivariable regression adjustment, propensity score weighting was used as a robust approach to reduce the effects of covariate confounding when estimating the adjusted odds of AKI.

**Results:**

A total of 228 patients were evaluated. The overall incidence of AKI was 11.8 % (27 of 228 patients). AKI occurred in 4 of 101 patients in the vanc group (4.0 %), 4 of 26 patients in the piptazo group (15.4 %), and 19 of 101 patients in the combV/P group (18.8 %). The univariable odds of AKI was significantly lower in the vanc group compared to both the combV/P group (OR 0.178, 95 % CI 0.058–0.544, p = 0.003) and piptazo (OR 0.227, 95 % CI 0.053–0.978, p = 0.047) group. A multivariable model accounting for baseline characteristics again showed that vanc monotherapy was associated with lower odds of AKI than combV/P (OR 0.14, 95 % CI 0.04–0.52, p = 0.004). Male sex was also associated with lower odds of AKI (OR 0.28, 95 % CI 0.10–0.79, p = 0.02) in the multivariable model. In the propensity score analysis using inverse probability of treatment weighting (IPTW), vanc monotherapy and male sex were again associated with lower odds of AKI (OR 0.17; 95 % CI 0.04–0.62, p = 0.008 and OR 0.28, 95 % CI 0.09–0.89, p = 0.03, respectively).

**Conclusion:**

This study substantiates recent reports that combV/P may be more associated with AKI than vanc monotherapy in hospital inpatients. AKI also appears to be more likely in females during therapy with these antimicrobials. While severity of illness is difficult to account for, these findings are further justification for narrowing antibiotic coverage when possible after this combination has been initiated in hospitalized patients.

## Background

The administration of early broad spectrum antibiotic therapy is important in the management of common infectious syndromes such as sepsis and health-care associated pneumonia [1]. As a result, point prevalence rates are high for antibiotic use in hospitalized patients [2]. However, antibiotics are sometimes used inappropriately and are associated with multiple adverse effects [3–5].

Acute kidney injury (AKI) during hospitalization occurs in approximately 5–20 % of inpatients and is an independent risk factor for mortality [6, 7]. AKI in this setting may occur in association with pharmaceutical agents such as intravenous contrast agents, non-steroidal anti-inflammatory drugs (NSAIDs), anticancer drugs, and antimicrobials [7].

Two antibiotics that are commonly used in hospitalized patients are vancomycin (vanc) for gram positive coverage and piperacillin/tazobactam (piptazo) for gram negative coverage. These two antimicrobials are often used in combination [8]. Given that nephrotoxicity has been associated with each of these antimicrobials [9, 10], we sought to determine if the risk of AKI is higher during combination vanc + piptazo therapy (combV/P) than during monotherapy with either agent in non-critically ill hospitalized patients. While studies focusing on intensive care unit (ICU) patients suggest that AKI may be associated with comb V/P, ICU patients have many confounders that increase their risk for AKI, including hypotension and shock [11]. Similar evidence from the non-critically ill hospitalized population has been heretofore lacking but is needed given that typically less than 10 % of hospitalized patients are admitted to intensive care units [12].

## Methods

We performed a retrospective study using the electronic medical record. Adult patients who were initiated on vanc and/or piptazo from January 2011 to March 2013 at Grady Health System (GHS), an urban academic medical center in Atlanta, were screened. Patients were excluded for the following reasons: (1) admission/transfer to intensive or intermediate care unit, (2) antibiotic use <48 h (subjects who experienced AKI prior to 48 h but antibiotics were continued beyond this timepoint were eligible for inclusion), (3) age <18 years, or (4) hemodialysis prior to therapy. AKI was defined by at least one of the two following: (1) ≥1.5-fold creatinine increase from admission baseline. (2) Increase in serum creatinine ≥0.5 mg/dl from admission baseline. Criterion 1 represents the lowest level of kidney injury defined by the RIFLE (risk, injury, failure, loss, end-stage kidney disease) scoring system, which has been used in multiple studies and shown to be associated with in-hospital mortality [13, 14]. Criterion 2 has been proposed as a sensitive measure of kidney injury that also allows for detection in patients with higher baseline creatinine levels yet remains associated with length of stay and mortality [6]. An exploratory analysis was also performed with the most inclusive measure of AKI we could find (serum creatinine increase ≥0.3 mg/dl as defined by the Acute Kidney Injury Network) [14, 15].

**Fig. 1:**
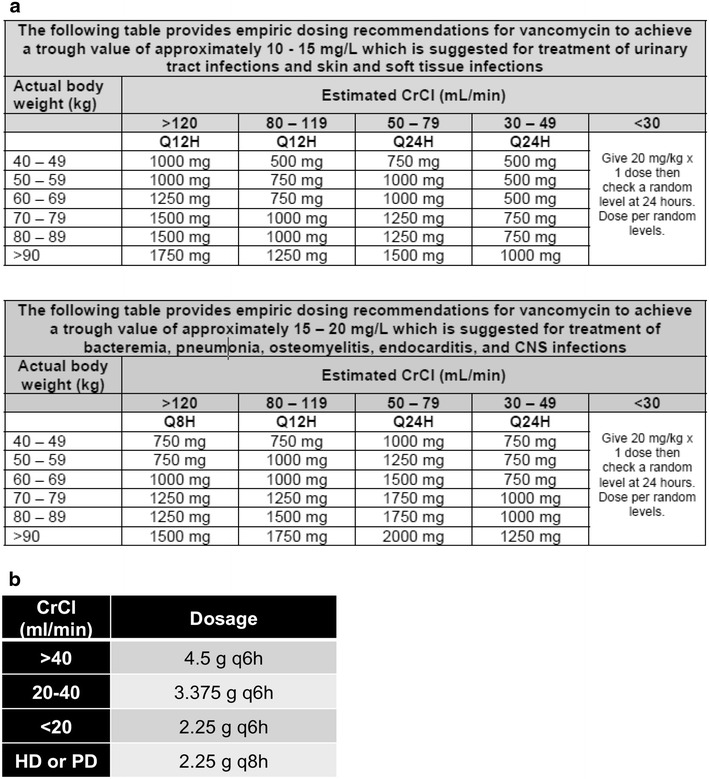
**a** GHS Vancomycin dosing guideline. **b** GHS Piperacillin–tazobactam dosing guideline

The primary outcome was the difference in AKI incidence between patients receiving vanc, piptazo or combV/P. Secondary outcomes included examination of risk factors for AKI. Concomitant nephrotoxic agents (defined as use within 24 h of vanc or piptazo) were intravenous (IV) contrast, aminoglycosides, amphotericin, NSAIDs and tenofovir. The following comorbidities were included: diabetes mellitus, hypertension, human immunodeficiency virus (HIV) infection, malignancy, and chronic kidney disease (CKD). CKD was defined as GFR <60 and was calculated automatically via the Modification of Diet in Renal Disease (MDRD) equation. Vanc and piptazo were dosed according to a hospital nomogram (Fig. 1).The study was approved by the Emory University Institutional Review Board and Grady Memorial Hospital Research Oversight Committee. As this was a retrospective study, we obtained a formal waiver of subject consent from both oversight groups.

### Statistical analysis

As per published literature, we estimated an inpatient AKI incidence of 10 % [6, 7]. With a rate of 10 %, a sample size of 100 non-critically ill hospitalized patients in each group produced a two-sided 95 % confidence interval with a width approximately 12 %. Additionally a sample size of 100 patients per antibiotic group (comb V/P versus monotherapy) provided 88 % power to detect a difference of 15 % in the proportion of patients with inpatient AKI (two-sided Fisher’s exact test; significance level = 0.05). This power calculation assumed that 20 % of patients receiving comb/V/P developed AKI and that there was a reduction to 5 % in the monotherapy groups. Data analysis was performed using SAS software. The potential associations with AKI were evaluated using the Chi square test or Fisher’s exact test. Baseline patient characteristics and risk factors significant to at least a P ≤ 0.10 were used in multiple logistic regression analysis.

Bootstrap bagging was used to identify stable and reliable predictors of AKI [16]. In brief, 1000 data sets were obtained by random sampling with replacement, automated forward stepwise regression was performed, and variables with *p* < 0.05 were identified. After aggregation of all analyses, variables appearing in 50 % or more of them were selected as reliable associations. In the final model, the odds ratio and its 95 % confidence interval were calculated for each factor in the presence of the others.

In addition, we performed a propensity score analysis incorporating the two groups with significant numbers of patients (vanc and combV/P groups). This was undertaken in order to account for treatment selection bias (i.e., systematic differences in clinical characteristics between patients in the two treatment groups that may affect treatment selection). Propensity scores or balancing scores were estimated using binary logistic regression with Vanc (vs combV/P) as the dependent variable. The independent variables for the propensity score model included covariates associated with treatment, outcome, and treatment and outcome. Covariates were included when the presence of the variable was found to be different in the univariate analysis between the two antibiotic groups with p value ≤0.1. Additionally, covariates were included that were found to have significant reliability >50 % in the first multivariable model with bootstrapping. The propensity or probability of receiving a particular treatment was calculated for each patient conditional on the covariates until an optimal balance on these covariates was achieved. To adjust for treatment selection bias, each patient was assigned a “weight” or influence when estimating the effect of treatment on acute kidney injury. The weight for each patient was inversely proportional to the probability of receiving the treatment to which they were assigned in reality (IPTW, inverse probability of treatment weighting). The weights were used in a propensity score-weighted logistic regression to determine the effect of treatment on AKI. Standardized differences were used to assess the balance of confounders between the two treatment groups using methods described by Austin [17]. A standardized difference less than 0.1 suggests negligible difference in the mean or prevalence of a covariate between treatment groups.

## Results

A total of 1172 patients were reviewed. 944 patients met exclusion criteria (586 patients were excluded due to ICU or intermediate care unit transfer, 353 patients received antibiotics for less than 48 h, and 5 patients were excluded due to hemodialysis receipt).Therefore, a total of 228 patients were identified after review of 26 months of inpatient admissions: 101 patients on vanc monotherapy, 26 patients on piptazo monotherapy, and 101 patients on combV/P. Baseline patient characteristics including age, gender, race, renal function in creatinine clearance based on Cockcroft and Gault equation (CrCl), and body mass index (BMI) are listed in Table 1. The majority of patients were male (64.9 %) and African-American (79.8 %). Co-morbidities were common (39.9 % had hypertension, 21.9 % had HIV, and 21.5 % had diabetes). The most common indications for antibiotic usage were skin/soft tissue infection followed by pneumonia, bone or joint infection, and sepsis of unknown etiology. Piptazo doses were appropriate according to renal function in >90 % of cases and vancomycin doses were greater than 4 g daily in <5 % of cases. Median time of antibiotic exposure for the patients with AKI was 68, 61 and 46 h for vanc, piptazo and combV/P groups respectively (all p > 0.05 between groups).

**Table 1:** Baseline patient characteristics

Characteristic^a^	N (%) [Mean, SD]
Years of age	[48.7, 15.3]
Male sex	148 (64.9)
Race	
African American	182 (79.8)
Caucasian	34 (14.9)
Hispanic	6 (2.6)
Native Hawaiian	1 (0.4)
Asian	2 (0.9)
Native American	2 (0.9)
Other	1 (0.4)
CrCl (ml/min) (n = 187)	[74.3, 32.8]
BMI (n = 187)	[26.8, 9.2]
Nephrotoxic agents	
IV contrast	50 (21.9)
Aminoglycoside	8 (3.5)
NSAID	31 (13.7)
Tenofovir	13 (5.7)
2 or more agents	17 (7.5)
Comorbidities	
Diabetes	49 (21.5)
Hypertension	91 (39.9)
Malignancy	16 (7.0)
CKD	12 (5.3)
HIV	50 (21.9)
2 or more of the above comorbidities	65 (28.5)
Indication for antibiotics	
Sepsis unknown etiology	26 (11.8)
Pneumonia	44 (19.3)
Bacteremia	7 (3.1)
Skin/soft tissue infection	76 (33.3)
Intra-abdominal	20 (8.8)
Central nervous system	2 (0.9)
Urinary tract	9 (4.0)
Bone/joint	41 (18.0)

*SD* standard deviation

^a^n = 228 unless otherwise stated

The overall incidence of AKI in the study was 11.8 % (27 of 228 patients). AKI occurred in 4 of 101 patients in the vanc group (4.0 %), 4 of 26 patients in the piptazo group (15.4 %), and 19 of 101 patients in the combV/P group (18.8 %). None of the patients required renal replacement therapy due to AKI. However, there were significant differences between the antibiotic treatment groups in demographic characteristics (Table 2). Patients receiving piptazo monotherapy were less likely to be African-American (61.5 %) than those on vanc monotherapy (83.2 %, p = 0.02) or combV/P (81.2 %, P = 0.03). Patients receiving piptazo monotherapy were more likely to be on concomitant NSAID therapy (34.6 %) than those on vanc monotherapy (13.0 %, p = 0.01) or combV/P (8.9 %, P = 0.001). Other nephrotoxic agents that were examined (intravenous contrast, tenofovir, and aminoglycosides) were not used at significantly different rates between antibiotic groups. None of the patients in the study received other agents known to be associated with nephrotoxicity including amphotericin and calcineurin inhibitors.

**Table 2:** Baseline characteristics by antibiotic

Baseline patient characteristic	Vancomycin (N = 101)	Piperacillin–tazobactam (N = 26)	Combination (N = 101)	p value (vanc vs piptazo)	p value (vanc vs combV/P)	p value (piptazo vs combV/P)	p value (overall)
Age: mean (sd)	47.00 (15.85)	47.58 (13.22)	50.71 (15.11)				0.21
Female sex (%)	39 (38.61 %)	9 (34.62 %)	32 (31.68 %)				0.59
African American (%)	84 (83.17 %)	16 (61.54 %)	82 (81.19 %)	0.02*	0.71	0.03*	0.04
CrCl in ml/min: mean (sd)	79.61 (36.35)	75.33 (36.12)	68.22 (26.70)				0.08
Number of nephrotoxic agents							0.98
0 agent	63 (63.00 %)	17 (65.38 %)	64 (63.37 %)				
1 agent	29 (29.00 %)	6 (23.08 %)	31 (30.69 %)				
2 agents	6 (6.00 %)	3 (11.54 %)	6 (5.94 %)				
3 agents	2 (2.00 %)	0	0				
Number of comorbidities				0.26	0.0012*	0.0026*	0.0006
0	46 (45.54 %)	16 (61.54 %)	26 (25.74 %)				
1	34 (33.66 %)	5 (19.23 %)	36 (35.64 %)				
2	17 (16.83 %)	4 (15.38 %)	34 (33.66 %)				
3	4 (3.96 %)	1 (3.85 %)	5 (4.95 %)				
BMI: mean (sd)	26.75 (9.72)	30.27 (10.05)	26.07 (8.23)				0.20
IV contrast	23 (22.77 %)	3 (11.54 %)	24 (23.76 %)				0.39
Aminoglycoside	5 (4.95 %)	0	3 (2.97 %)				0.44
NSAID	13 (13.00 %)	9 (34.62 %)	9 (8.91 %)	0.0097*	0.35	0.0008*	0.003
Tenofovir	6 (5.94 %)	0	7 (6.93 %)				0.39
Diabetes	16 (15.84 %)	3 (11.54 %)	30 (29.70 %)	0.58	0.02*	0.06	0.02
Hypertension	32 (31.68 %)	10 (38.46 %)	49 (48.51 %)	0.51	0.01*	0.36	0.05
Malignancy	5 (4.95 %)	2 (7.69 %)	9 (8.91 %)				0.54
CKD	5 (4.95 %)	0	7 (6.93 %)				0.36
HIV	22 (21.78 %)	1 (3.85 %)	27 (26.73 %)	0.03*	0.41	0.01*	0.04
Indication for antibiotics							
Pneumonia	15 (14.85 %)	1 (3.85 %)	28 (27.72 %)	0.1899	0.03*	0.0097*	0.0072
bacteremia	5 (4.95 %)	0	2 (1.98 %)				0.46
SSTI	40 (39.60 %)	4 (15.38 %)	32 (31.68 %)				0.06
Abdominal	1 (0.99 %)	15 (57.69 %)	4 (3.96 %)	<0.0001*	0.37	<0.0001*	<0.0001
CNS	2 (1.98 %)	0	0				0.61
UTI	3 (2.97 %)	5 (19.23 %)	1 (0.99 %)	0.0091*	0.62	0.0013*	0.0003
Bone or joint infection	25 (24.75 %)	0	16 (15.84 %)	0.0046*	0.11	0.04*	0.01
Sepsis of unknown etiology	10 (10.20 %)	1 (4.55 %)	15 (15.00 %)				0.31

P < 0.05 between individual groups denoted by italics and asterisk

Overall, patients on vanc or piptazo monotherapy had fewer cumulative comorbidities than those on combV/P. Specifically, patients on vanc were less likely to have hypertension (p = 0.01) and diabetes (p = 0.02) compared to patients on combV/P. Patients on piptazo were less likely to have HIV than patients on vanc (p = 0.03) or combV/P (p = 0.01). Not surprisingly, the indications for antibiotics were also significantly different between groups. Compared to patients on vanc and combV/P, patients on piptazo were significantly more likely to have urinary tract infection and intra-abdominal infection but significantly less likely to have bone/joint infection. Patients on combV/P were significantly more likely to have pneumonia than patients on vanc or piptazo monotherapy (all P < 0.05).

In the univariable analysis (Table 3), the odds of AKI were lower in the vanc group compared to both the comb V/P group (OR 0.178, p = 0.003) and to the piptazo monotherapy group (OR 0.227, p = 0.047). There was no significant difference in AKI between the piptazo and combV/P groups. For the exploratory analysis using the most conservative measure of AKI (creatinine increase of at least 0.3 mg/dl), the frequency was again higher in the combV/P group (26 of 101 or 25.7 %) compared to the vanc monotherapy group (11 or 101 or 10.9 %, p = 0.006). Also in this exploratory analysis, AKI occurred in 6 of 26 patients (23.1 %) of the piptazo group, which was not statistically different compared to the vanc group (p = 0.10) or compared to the combV/P group (p = 0.78).

**Table 3:** Univariable analysis for AKI [27 of 228 (11.8 %) patients with AKI]

Risk factor	Incidence AKI	% with AKI	Odds ratio	95 % CI	P value
Treatment					
Vancomycin	4/101	4.0	0.178	(0.058, 0.544)	0.003*
Piperacillin/tazobactam	4/26	15.4	0.785	(0.242, 2.545)	0.69
Combination vanc + piptazo	19/101	18.8	1.0		
Vanc			0.227	(0.053, 0.978)	0.047*
Piptazo			1.0		
Age					
Per 1 year increase			0.994	(0.968, 1.021)	0.69
Sex					
Male	11/148	7.4	0.321	(0.141, 0.731)	0.007*
Female	16/80	20.0			
Race					
Caucasian	1/34	2.2	1.0		
African-American	26/182	14.3	5.5	(0.721–41.968)	0.10
BMI					
≥30 kg/m^2^	9/43	20.9	2.667	(1.053, 6.760)	0.04*
<30 kg/m^2^	13/144	9.0	1.0		
Creatinine clearance					
<Median of 71.94 ml/min	11/95	11.6	0.964	(0.396, 2.347)	0.94
≥Median of 71.94 ml/min	11/92	12.0	1.0		
Per 10 ml/min decrease			1.048	(0.922, 1.192)	0.47
Nephrotoxic agents					
IV contrast, yes	8/50	16.0	1.594	(0.652, 3.894)	0.31
IV contrast, no	19/178	10.7	1.0		
Aminoglycoside, yes	1/8	12.5	1.066	(0.126, 9.014)	0.95
Aminoglycoside, no	26/220	11.8	1.0		
NSAIDs, yes	5/31	16.1	1.521	(0.530, 4.368)	0.44
NSAIDs, no	22/196	11.2	1.0		
Tenofovir, yes	2/13	15.4	1.382	(0.290, 6.599)	0.68
Tenofovir, no	25/215	11.6	1.0		
≥2 agents	1/17	5.9	0.445	(0.057, 3.495)	0.44
<2 agents	26/211	12.3	1.0		
Diabetes					
Yes	7/49	14.3	1.325	(0.525, 3.343)	0.55
No	20/179	11.2	1.0		
Hypertension					
Yes	14/91	15.4	1.734	(0.774, 3.886)	0.18
No	13/137	9.5	1.0		
Malignancy					
Yes	3/16	18.8	1.808	(0.480, 6.803)	0.38
No	24/212	11.3	1.0		
CKD					
Yes	2/12	16.7	1.528	(0.317, 7.377)	0.60
No	25/216	11.6	1.0		
HIV					
Yes	6/50	12.0	1.020	(0.388, 2.681)	0.97
No	21/178	11.8	1.0		

P values <0.05 denoted by italics face plus asterisk

Table 3 shows demographic variables that by univariable analysis (p ≤ 0.10 for inclusion in multivariable model) were associated with increased or decreased odds of AKI: male gender, OR 0.321 (95 % CI 0.141–0.731, p = 0.007), BMI ≥30 kg/m^2^, OR = 2.667 (95 % CI 1.053–6.760, p = 0.04), and African-American race compared to caucasian OR 5.5 (95 % CI 0.721–41.968 p = 0.10). Of the indications for antibiotics (not listed in Table 3), pneumonia (OR 3.50, 95 % CI 1.491–8.219, p = 0.004) was associated with higher odds of AKI while skin/soft tissue infection (OR 0.137, 95 % CI 0.032–0.596, p = 0.008) was associated with a lower odds for AKI. Intra-abdominal infection (OR 2.819, 95 % CI 0.934–8.504) tended to have higher odds of AKI (p = 0.07).

For the traditional multivariable analysis with logistic regression, there were 179 patients, 22 with AKI (see Table 4). By bootstrap bagging, the following factors occurred in over 50 % of models and were thus kept in the model: antibiotic group (84.1 %), sex (67.4 %), baseline creatinine clearance (per 10 ml/min increase) (65.4 %), African-American versus caucasian race (57.3 %), and skin/soft tissue infection (52.1 %). The other factors from Tables 2 and 3 with p ≤ 0.10 occurred in <50 % of models and thus were not kept in the model. In the final model, vanc monotherapy (OR 0.14, 95 % CI 0.04–0.52, p = 0.004) as well as piptazo monotherapy (OR 0.15, 95 % CI 0.03–0.83, p = 0.03) were associated with lower odds of AKI compared to comb V/P. Male sex was also associated with lower odds of AKI (OR 0.28, 95 % CI 0.10–0.79, p = 0.02). There was also a trend for increasing creatinine clearance to be associated with higher odds of AKI (p = 0.05) and for skin/soft tissue infection to be associated with lower odds of AKI (p = 0.06).

**Table 4:** Multivariable logistic regression analysis for factors associated with AKI (179 patients and 22 with AKI)

Risk factor	Odds ratio (95 % CI)	P	Reliability^a^ (%)
Antibiotic group		0.01	84.6
Vanc versus combV/P	0.14 (0.04, 0.52)	0.004	
Piptazo versus combV/P	0.91 (0.22, 3.82)	0.89	
Vanc versus piptazo	0.15 (0.03, 0.83)	0.03	
Sex (male/female)	0.28 (0.10, 0.79)	0.02	67.4
Baseline creatinine clearance (per 10 ml/min increase)	1.15 (1.00, 1.33)	0.05	65.4
Race (African-American/Caucasian)	5.34 (0.62, 46.4)	0.13	57.3
Skin/soft tissue infection (yes/no)	0.22 (0.05, 1.05)	0.06	52.1

Risk factors with reliability less than 50 % included pneumonia (48 %), hypertension (33 %), BMI (28 %), NSAIDS (25 %), bone or joint infection (19 %), diabetes (13 %), HIV (11 %), and total number of comorbidities (10 %)

^a^Percentage of times each risk factor appeared in 1000 bootstrap multivariable analyses. Risk factors with reliability <50 % were not included in the multivariable model

Given the very small number of patients in the piptazo group, we henceforth focused the propensity score logistic regression analysis on only the vanc and combV/P groups. The significant covariates from Tables 2 and 4 included race, baseline creatinine clearance, pneumonia, diabetes, hypertension and skin/soft tissue infection. We did not include subjects who lacked creatinine clearance data in the propensity score analysis, leaving 161 subjects to be analyzed by IPTW (see Table 5). Vanc monotherapy again was associated with a decreased odds ratio for AKI compared to combV/P (adjusted OR 0.17; 95 % CI 0.04–0.62, p = 0.008). Male sex was also again associated with decreased odds ratio for AKI (adjusted OR 0.28, 95 % CI 0.09–0.89, p = 0.03). There were no other statistically significant associations with AKI in the propensity score analysis.

**Table 5:** Logistic regression with inverse probability of treatment weighting to address treatment selection bias (n = 161)

Risk factor	Odds ratio (95 % CI)	P
Antibiotic group		
Vanc versus combV/P	0.17 (0.04, 0.62)	0.008
Sex (male/female)	0.28 (0.09, 0.89)	0.03
Baseline creatinine clearance (per 10 ml/min increase)	1.14 (0.97, 1.34)	0.11
Race (African-American/Caucasian)	5.67 (0.47, 68.40)	0.17
Skin/soft tissue infection (yes/no)	0.49 (0.11, 2.25)	0.36
Pneumonia (yes/no)	2.25 (0.63, 8.07)	0.21
Diabetes (yes/no)	0.77 (0.19, 3.17)	0.71
Hypertension (yes/no)	1.78 (0.55, 5.78)	0.34

## Discussion

Given the high frequency of infections in hospitalized patients caused by drug resistant pathogens, empiric broad spectrum antibiotic use will be common for the foreseeable future. In this retrospective study of non-critically ill inpatients, we found that compared to vanc monotherapy, the odds of AKI were over five times higher during combV/P therapy. We also found that odds of AKI were significantly lower for males during therapy with these antibiotics. These associations remained statistically significant in the multivariable analyses that included propensity scoring.

At the time of this study, there was a paucity of published data examining the incidence of AKI with combV/P therapy versus monotherapy with either agent, particularly in hospitalized patients who are not critically ill [18, 19]. Since we completed our analysis, however, two studies were published in 2014 that focused on this question in non-critically ill hospitalized patients [20, 21]. Each study incorporated definitions of AKI similar to ours and both also showed that combV/P was independently associated with AKI compared with vanc monotherapy. In the study by Burgess et al., 191 patients who had received treatment with vancomycin were analyzed. In a multivariable analysis, comb V/P was associated with an AKI odds ratio of 2.48 (p = 0.032) compared to vanc monotherapy. In the study by Meaney et al., 125 patients treated with vancomycin were analyzed. Again, comb V/P was associated with an AKI odds ratio of 5.36 (95 % CI 1.41–20.5) compared to vanc monotherapy in multivariate analyses. Our study provides further proof of this association and extends the finding to a setting in which patients are predominantly African-american. Our study provides additional evidence through a propensity score analysis that shows the same statistically significant AKI odds ratio associated with combV/P. While a randomized controlled trial would be ideal to evaluate such risks, propensity score analysis can provide an estimate of the likelihood of exposure based on demographics and other co-morbidities.

The mechanisms that could underpin AKI during combV/P are not clear at this time. Beta lactam agents, in particular penicillin derivatives, are purported to cause AKI through interstitial nephritis [22]. Interestingly, the study by Meaney et al. found that almost one-third of patients with AKI also had either eosinophilia or eosinophiluria. This may be additional evidence that AKI on combV/P may be in part immune mediated. It should be noted that there are conflicting reports on whether combV/P is more associated with AKI than the combination of vancomycin and cefepime and other cephalosporins [23, 24]. Therefore, AKI during combination therapy with vancomycin and beta-lactams may or may not be a class effect. Larger prospective studies are needed to more definitively address this question.

Our multivariable analysis showed that female patients were more likely to develop AKI during treatment with at least one of the two antimicrobials in the study. To our knowledge this finding has not been previously reported, but female gender has been identified as a risk factor for AKI in the most recent Kidney Disease Improving Global Outcomes (KDIGO) statement [25]. The mechanism for this is unclear, but based on our study clinicians should be aware that female inpatients may be at higher risk for AKI during treatment with vanc or piptazo. Unexpectedly, we found a trend for increased AKI odds with increasing creatinine clearance (by 10 ml/min increase). This association is counterintuitive but has also been found in at least two other studies of vancomycin nephrotoxicity which also used the Cockroft-Gault (CG) equation for estimate of renal function [21, 26]. Both antibiotics are dosed renally meaning that patients with higher CrCl are often given a higher dose of drug. This may have played a role in this finding. However, this association became statistically non-significant in the propensity score analysis. We also acknowledge that the CG equation is not considered the gold standard for measurement of renal function and has variability based on body surface area [27]. It would be ideal to measure glomerular filtration rate directly in order to gauge renal function, but the feasibility of accomplishing this in a large study is significantly lower.

We acknowledge the other limitations of our study. Propensity scoring is one way to compare the risks of exposure to drug (and thus can in some ways account for demographic and disease differences), but is not a perfect substitute for a randomized trial. For example, the lower odds of AKI associated with skin/soft tissue infection could be a reflection of less severe illness and may have influenced the finding of lower AKI odds in the vanc monotherapy group. While we attempted to account for illness severity in the multivariable analysis and propensity score analysis by including comorbidities and indication for antibiotics, we were not able to perform analyses of illness severity as measured by scores such as APACHE (Acute Physiologic Assessment and Chronic Health Evaluation) and other equations [28]. Ideally, prospective studies are necessary to address causality and a randomized clinical trial would be the most rigorous means to determine a causal treatment effect.

We also acknowledge the lack of collection of some data points, including duration of hospitalization. We also were not able to evaluate the potential role of vancomycin concentrations in this study of AKI. While very few patients in the group as a whole were given vancomycin dosing greater than the 4 g daily threshold that has been shown to be associated with AKI, we were not able to specifically analyze whether this was a risk factor for AKI at an individual level. We also did not analyze the data with respect to the companies that provided the drugs over the course of the study.

We did not assess the reversibility of AKI over time, but did find that there were no patients who required renal replacement therapy during the hospitalization. As with other recently published studies, we excluded patients who received antibiotics for less than 48 h [20, 21]. However, other study designs may have been appropriate, such as including all patients who had at least one dose of antibiotic. For the definition of AKI in our study, we chose to include the lowest level of injury defined by the RIFLE criteria as well as an additional criterion that has been advocated by some experts to define AKI. It is possible that the use of these two criteria was overly inclusive and that the results may have been different if a more stringent definition of AKI had been used. However, we believe that an inclusive definition was appropriate for this study given that even low levels of renal injury have been associated with poor clinical outcomes in hospitalized patients [14]. It is also possible that our definition of AKI was not inclusive enough. However, an exploratory analysis using a particularly inclusive measure of AKI also showed increased AKI in those on combV/P compared to vanc monotherapy.

## Conclusions

In summary, in a sample of non-critically ill inpatients, we found increased odds for acute kidney injury associated with combination vancomycin + piperacillin/tazobactam compared to vancomycin monotherapy. Additionally, we found female sex to be independently associated with AKI during therapy with these antimicrobials. The associations remained significant in robust multivariable analyses and propensity score analyses. These findings provide further justification for narrowing antibiotic coverage when possible after this combination has been initiated in hospitalized patients. Larger multicenter prospective studies are needed to confirm these findings.

## Abbreviations

AKI: acute kidney injury
vanc: vancomycin
piptazo: piperacillin/tazobactam
combV/P: combination vancomycin + piperacillin–tazobactam
NSAID: non-steroidal anti-inflammatory drug
GHS: Grady Health System
RIFLE: risk, injury, failure, loss, end-stage kidney disease
CKD: chronic kidney disease
HIV: human immunodeficiency virus
CKD: chronic kidney disease
MDRD: modification of diet in renal disease
CrCl: creatinine clearance
BMI: body mass index
OR: odds ratio
KDIGO: Kidney Disease Improving Global Outcomes
CG: cockroft-gault
APACHE: Acute Physiologic Assessment and Chronic Health Evaluation

## References

[1] Kumar A, Ellis P, Arabi Y, Roberts D, Light B, Parrillo JE, Dodek P, Wood G, Kumar A, Simon D (2009). Initiation of inappropriate antimicrobial therapy results in a fivefold reduction of survival in human septic shock. Chest.

[2] Ansari F, Erntell M, Goossens H, Davey P (2009). The European surveillance of antimicrobial consumption (ESAC) point-prevalence survey of antibacterial use in 20 European hospitals in 2006. Clin Infect Dis Off Publ Infect Dis Soc Am.

[3] Ingram PR, Seet JM, Budgeon CA, Murray R (2012). Point-prevalence study of inappropriate antibiotic use at a tertiary Australian hospital. Intern Med J.

[4] John JF, Fishman NO (1997). Programmatic role of the infectious diseases physician in controlling antimicrobial costs in the hospital. Clin Infect Dis Off Publ Infect Dis Soc Am.

[5] Polgreen PM, Chen YY, Cavanaugh JE, Ward M, Coffman S, Hornick DB, Diekema DJ, Herwaldt LA (2007). An outbreak of severe Clostridium difficile-associated disease possibly related to inappropriate antimicrobial therapy for community-acquired pneumonia. Infect Control Hosp Epidemiol Off J Soc Hosp Epidemiol Am.

[6] Chertow GM, Burdick E, Honour M, Bonventre JV, Bates DW (2005). Acute kidney injury, mortality, length of stay, and costs in hospitalized patients. J Am Soc Nephrol JASN.

[7] Hou SH, Bushinsky DA, Wish JB, Cohen JJ, Harrington JT (1983). Hospital-acquired renal insufficiency: a prospective study. Am J Med.

[8] Cometta A, Kern WV, De Bock R, Paesmans M, Vandenbergh M, Crokaert F, Engelhard D, Marchetti O, Akan H, Skoutelis A (2003). Vancomycin versus placebo for treating persistent fever in patients with neutropenic cancer receiving piperacillin–tazobactam monotherapy. Clin Infect Dis Off Publ Infect Dis Soc Am.

[9] Elyasi S, Khalili H, Dashti-Khavidaki S, Mohammadpour A (2012). Vancomycin-induced nephrotoxicity: mechanism, incidence, risk factors and special populations. A literature review. Eur J Clin Pharmacol.

[10] Murray KM, Keane WR (1992). Review of drug-induced acute interstitial nephritis. Pharmacotherapy.

[11] Ostermann M, Chang RW (2007). Acute kidney injury in the intensive care unit according to RIFLE. Crit Care Med..

[12] Groeger JS, Strosberg MA, Halpern NA, Raphaely RC, Kaye WE, Guntupalli KK, Bertram DL, Greenbaum DM, Clemmer TP, Gallagher TJ (1992). Descriptive analysis of critical care units in the United States. Crit Care Med.

[13] Bellomo R, Ronco C, Kellum JA, Mehta RL, Palevsky P, Acute Dialysis Quality Initiative w (2004). Acute renal failure—definition, outcome measures, animal models, fluid therapy and information technology needs: the Second International Consensus Conference of the Acute Dialysis Quality Initiative (ADQI) Group. Crit Care.

[14] Van Biesen W, Vanholder R, Lameire N (2006). Defining acute renal failure: RIFLE and beyond. Clin J Am Soc Nephrol CJASN.

[15] Mehta RL, Kellum JA, Shah SV, Molitoris BA, Ronco C, Warnock DG, Levin A, Acute Kidney Injury N (2007). Acute Kidney Injury Network: report of an initiative to improve outcomes in acute kidney injury. Crit Care.

[16] Breiman L (1996). Bagging predictors. Mach Learn.

[17] Austin PC (2011). An introduction to propensity score methods for reducing the effects of confounding in observational studies. Multivar Behav Res.

[18] Hellwig T, Hammerquist, R. Critical Care Congress 2012 Poster 301: Retrospective evaluation of the incidence of vancomycin and/or piperacillin–tazobactam induced acute renal failure. Crit Care Med 39 (supplement 12). In: Houston, Texas; 2012.

[19] Min E, Box K, Lane J, Sanchez J, Coimbra R, Doucet J, Potenza B, Wargel L. Critical Care Congress 2012 Poster 714: Acute kidney injury in patients receiving concomitant vancomycin and piperacillin/tazobactam. Critical Care Medicine volume 79 (supplement 12). In: Houston, Texas; 2012.

[20] Burgess LD, Drew RH (2014). Comparison of the incidence of vancomycin-induced nephrotoxicity in hospitalized patients with and without concomitant piperacillin–tazobactam. Pharmacotherapy.

[21] Meaney CJ, Hynicka LM, Tsoukleris MG (2014). Vancomycin-associated nephrotoxicity in adult medicine patients: incidence, outcomes, and risk factors. Pharmacotherapy.

[22] Baldwin DS, Levine BB, McCluskey RT, Gallo GR (1968). Renal failure and interstitial nephritis due to penicillin and methicillin. N Engl J Med.

[23] Gomes DM, Smotherman C, Birch A, Dupree L, Della Vecchia BJ, Kraemer DF, Jankowski CA (2014). Comparison of acute kidney injury during treatment with vancomycin in combination with piperacillin–tazobactam or cefepime. Pharmacotherapy.

[24] Moenster RP, Linneman TW, Finnegan PM, Hand S, Thomas Z, McDonald JR (2014). Acute renal failure associated with vancomycin and beta-lactams for the treatment of osteomyelitis in diabetics: piperacillin–tazobactam as compared with cefepime. Clin Microbiol Infect Off Publ Eur Soc Clin Microbiol Infect Dis.

[25] Kidney Disease: Improving Global Outcomes (KDIGO) Acute Kidney Injury Work Group (2012). AKI definition. Kidney Int Suppl.

[26] Moh’d H, Kheir F, Kong L, Du P, Farag H, Mohamad A, Zurlo JJ (2014). Incidence and predictors of vancomycin-associated nephrotoxicity. South Med J.

[27] Shoker A, Hossain MA, Koru-Sengul T, Raju DL, Cockcroft D (2006). Performance of creatinine clearance equations on the original Cockcroft-Gault population. Clin Nephrol.

[28] Vincent JL, Ferreira F, Moreno R (2000). Scoring systems for assessing organ dysfunction and survival. Crit Care Clin.

